# Revealing the Angiopathy of Lacrimal Gland Lesion in Type 2 Diabetes

**DOI:** 10.3389/fphys.2021.731234

**Published:** 2021-08-31

**Authors:** Junfa Xue, Bin Zhang, Shengqian Dou, Qingjun Zhou, Min Ding, Mingming Zhou, Huifeng Wang, Yanling Dong, Dongfang Li, Lixin Xie

**Affiliations:** ^1^School of Medicine and Life Sciences, Shandong First Medical University, Jinan, China; ^2^State Key Laboratory Cultivation Base, Shandong Province Key Laboratory of Ophthalmology, Shandong Eye Institute, Shandong First Medical University & Shandong Academy of Medical Sciences, Qingdao, China; ^3^Qingdao Eye Hospital of Shandong First Medical University, Qingdao, China; ^4^Department of Medicine, Qingdao University, Qingdao, China

**Keywords:** diabetes, lacrimal gland, vasculature, DEGs, hub gene

## Abstract

For a better understanding of diabetic angiopathy (DA), the potential biomarkers in lacrimal DA and its potential mechanism, we evaluated the morphological and hemodynamic alterations of lacrimal glands (LGs) in patients with type 2 diabetes and healthy counterparts by color Doppler flow imaging (CDFI). We further established a type 2 diabetic mice model and performed hematoxylin-eosin (HE) staining, immunofluorescence staining of CD31, RNA-sequencing analysis, and connectivity map (CMap) analysis. We found atrophy and ischemia in patients with type 2 diabetes and mice models. Furthermore, we identified 846 differentially expressed genes (DEGs) between type 2 diabetes mellitus (T2DM) and vehicle mice by RNA-seq. The gene ontology (GO) analysis indicated significant enrichment of immune system process, regulation of blood circulation, apoptotic, regulation of secretion, regulation of blood vessel diameter, and so on. The molecular complex detection (MCODE) showed 17 genes were involved in the most significant module, and 6/17 genes were involved in vascular disorders. CytoHubba revealed the top 10 hub genes of DEGs, and four hub genes (App, F5, Fgg, and Gas6) related to vascular regulation were identified repeatedly by MCODE and cytoHubba. GeneMANIA analysis demonstrated functions of the four hub genes above and their associated molecules were primarily related to the regulation of circulation and coagulation. CMap analysis found several small molecular compounds to reverse the altered DEGs, including disulfiram, bumetanide, genistein, and so on. Our outputs could empower the novel potential targets to treat lacrimal angiopathy, diabetes dry eye, and other diabetes-related diseases.

## Introduction

Type 2 diabetes mellitus (T2DM) and diabetic complications have been with high mortality and morbidity year by year and arouse great concern throughout the globe (Vujkovic et al., 2020). Dry eye (DE) syndrome, as one of the main diabetic complications on the ocular surface, is a common eye disease due to a reduction in the volume or quality of tears (Nakamachi et al., 2016) manifesting as increased tear evaporation, hyperosmolarity, and tear film instability, as well as vulnerability to infection, inflammation, sensory neuropathy, and damage of the ocular surface (Markoulli et al., 2018). Tear components are secreted mainly from the lacrimal gland (LG), which has a high risk of lesion and secretion deficiency in patients affected by diabetes (Jiao et al., 2020; Qu et al., 2021). The possible reason for the lacrimal lesion in diabetes may be angiopathy for the dense vasculature distributions in LG and the critical role of angiopathy in the pathogenesis ofs diabetes, diabetic retinopathy, and diabetic nephropathy (Gao et al., 2019).

Previous studies have revealed the LG atrophied and morphology change in type 1 diabetes (Jiao et al., 2020; Qu et al., 2021). There is growing evidence that AGE, AGER, and immune-related molecules (like NF-κB, interferons, and interleukin-27) are highly expressed in LGs of a diabetic animal model (Alves et al., 2005; Ciecko et al., 2019; Allred et al., 2021), which proves that these factors are involved in signaling and subsequent inflammatory alterations related to DE in diabetes. A study by our research groups has demonstrated that the mitochondrial bioenergetic deficit in diabetic LG may contribute to the early onset of DE, while mitochondria-targeted antioxidant has therapeutic potential for diabetic DE and keratopathy (Qu et al., 2021). However, type 2 diabetes accounts for 90–95% of all diabetes morbidity (Basterra-Gortari et al., 2019), the lacrimal lesion of which has not been fully elucidated. The microvascular complication of diabetes generated in retina, kidney, and nervous system (Knudsen et al., 2019) has not been clearly illustrated in the LG.

**Figure 1:**
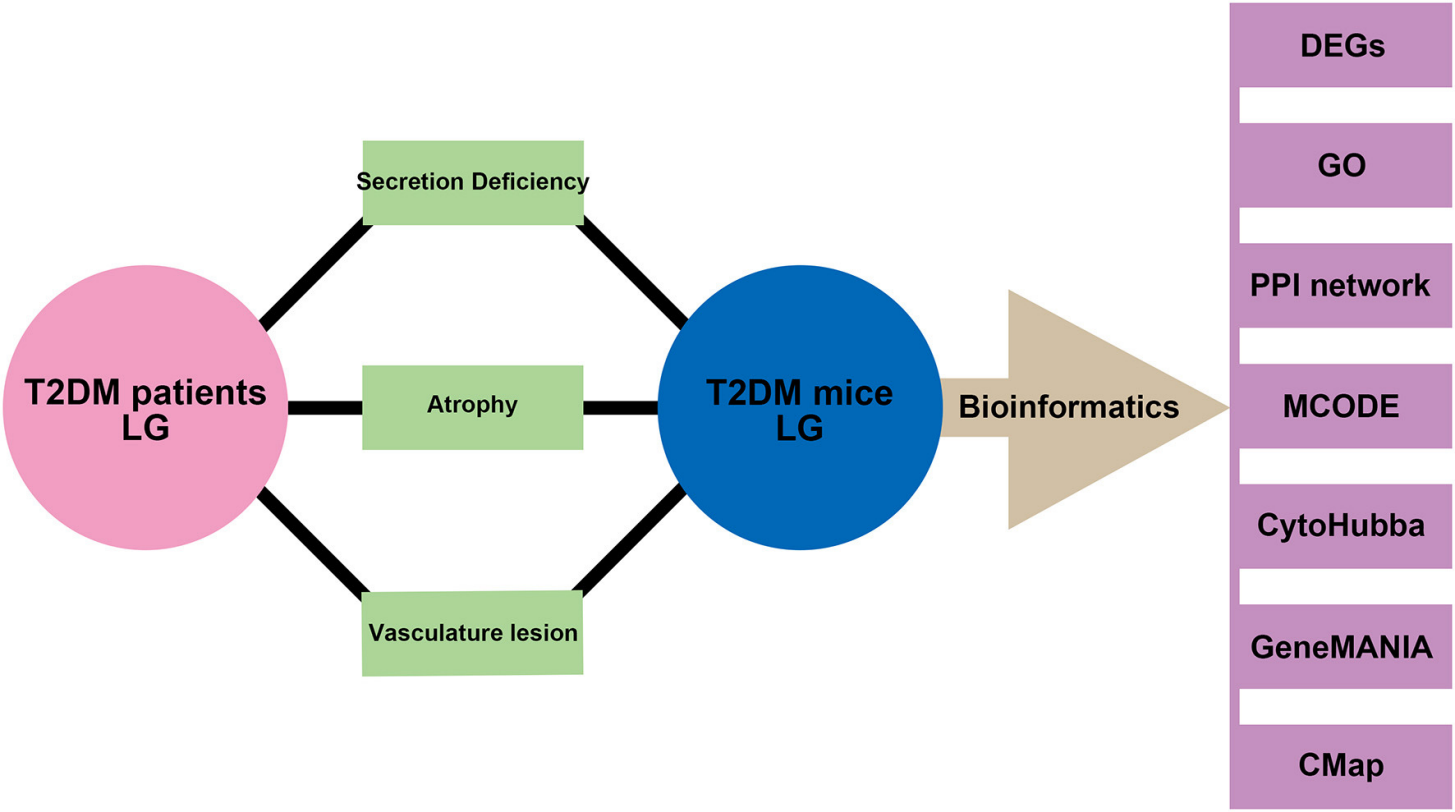
Schematic diagram of the study. Phenotypes of secretion deficiency, atrophy, and vascular lesion were both found in the lacrimal gland from patients with type 2 diabetes and mice model. Bioinformatics of samples from the mice model was performed to further identify the potential biomarkers and mechanisms.

In the present study, we aim to investigate the morphology and blood flow status changes in LG of patients with type 2 diabetes by color Doppler flow imaging (CDFI) (Bilgili et al., 2005; Lecler et al., 2017). To better understand the potential biomarkers and mechanisms for diabetic lacrimal lesion, lacrimal angiopathy, diabetes, and other metabolic diseases, we established the type 2 diabetic mice model and performed the RNA-sequencing. By using gene ontology (GO) analysis, protein-protein interaction (PPI) network, molecular complex detection (MCODE), cytoHubba, and geneMANIA, we aim to identify the hub biomarkers and their related mechanisms in diabetic lacrimal angiopathy. Furthermore, a connectivity map (CMap) analysis was performed to predict the potential therapeutic agents. The schematic diagram of the whole study is presented in Figure 1. Our output could empower the novel and more comprehensive diagnostic and therapeutic targets for type 2 diabetic lacrimal angiopathy, diabetic DE, and other chronic metabolic diseases.

## Materials and Methods

### Ethical Approval

#### Clinical Research Section

The single-center prospective clinical controlled trial study was approved by the Ethics Committee of Qingdao Eye Hospital (No. 2019-27) and adhered to the Declaration of Helsinki's tenets. All subjects agreed to participate in this study and signed the informed consent. The clinical trial was registered on the Chinese Clinical Trial Registry (www.chictr.org.cn), and the registration number is ChiCTR2000032843.

#### Basic Research Section

All studies on animals were approved by the Experimental Animal Ethics Committee of Shandong first medical University and were performed in accordance with the Association for Research in Vision and Ophthalmology (ARVO) Statement for the Use of Animals in Ophthalmic and Vision Research.

### Clinical General Information

Patients with type 2 diabetes were randomly selected from outpatients with a hemoglobin A1C value above 6.5% at Qingdao Eye Hospital. Thirty patients with diabetes for <10 years (average age, 59.00 ± 7.89 years), 30 patients with diabetes for more than 10 years (average age, 63.67 ± 9.00 years), and 30 controls with no history and signs of diabetes (average age, 60.97 ± 12.63 years) were enrolled and received examinations from June 2019 to June 2020. All of them had no history of ocular surgery or trauma, other systemic diseases, or utilization of drugs known to disturb blood flow. Female subjects were not in the menstrual period. All subjects lived in Shandong province for a long time (more than 10 years) and followed the diet habits of the Han nationality. All examinations were performed by a proficient operator (MZ).

### Clinical Examination Methods

All subjects underwent a general ophthalmologic examination, including the Schirmer test and CDFI. The results of one randomly chosen eye of each subject were assessed. No complications or complaints of orbital tissue discomfort due to the measurements occurred. Researchers were blind to the history of diabetes of enrolled subjects before examinations.

All procedures of CDFI were performed in strict accordance with the standard process and previous research methods (Bilgili et al., 2005; Lecler et al., 2017). The CDFI was performed when patients were supine with eyelids closed. The lacrimal region (superolateral margin of the orbit) was scanned obliquely, with a scanning plane almost parallel to the anterior outline of the orbit, and using part of the ocular globe as an acoustic window. The lacrimal artery has proved to be an optimal landmark to identify the gland (Bilgili et al., 2005). As the LG was identified, the scanning plane was oriented to view its longest diameter. Color Doppler sonography was then performed by the same operator with an ultrasound scanner (Esaote MyLab Class C Advanced, Italy) equipped with a multifrequency linear-array transducer (6–18 MHz). Doppler parameters were adjusted for detection of low velocity or low volume flow or both. The pulse repetition frequency used was 1.3 kHz, the bandpass filter was set at 50 Hz, and high levels were adjusted for both color vs. echo priority and color persistence. To further increase the depiction of vessel continuity, the power Doppler mode was also used. To evaluate the lacrimal artery by pulsed Doppler sonography, we used a pulse repetition frequency of 1.3 kHz, a sample volume of 0.5 mm, and gain 55%. Peak systolic velocity (PSV) was measured. Resistance index (RI) and pulsatility index (PI) values were calculated to evaluate peripheral resistance inside the gland. To avoid the excessively high temperature caused by increased energy necessary for Doppler, the procedure was limited for the minimum amount of time required to effectively evaluate the patient. All the LGs were scanned in two dimensions (longitudinal section of the LG and cross-section of the LG), and the images were processed by ImageJ to calculate the pixels of the lacrimal area in the same size of graphs.

### Animal Model

To establish the T2DM mice model (Xing et al., 2021), 4-week-old male C57BL/6 (B6) mice (purchased from the SPF Biotechnology Co., Ltd., Beijing, China, and fed in the Laboratory animal Center of Shandong Eye Institute) were fed with a high-fat diet (HFD, Cat#D12451, Beijing Keao Xieli Feed Co., Ltd., China; 60 kcal% fat, HFD) for 6 weeks. The mice were then fasted and intraperitoneal injection of a low dose of streptozotocin (STZ) of 50 mg/kg (Sigma-Aldrich, St. Louis, MO) for 5 consecutive days, then kept on an HFD diet for the next 24 weeks. The control mice were fed with a normal diet and injected with citrate buffer only. Diabetic mice were defined as having instant blood glucose levels > 16.7 mmol/L. Experiments *in vivo* and *vitro* were tabbed by one assistant and performed by another operator.

### Histopathology and Immunofluorescence Staining

Paraffin sections of LGs were stained with hematoxylin and eosin. Immunohistochemistry of amyloid precursor protein (App; ab32136, Abcam) and fibrinogen gamma chain (Fgg; ab281924, Abcam) was performed following standard procedures. According to a previous study (Cao et al., 2011), the whole-mount immunofluorescence staining of LGs was collected and fixed in 4% paraformaldehyde in 0.1 M phosphate saline (Phosphate Buffered Saline, pH 7.2–7.4) for 45 min at room temperature. The samples were then washed in PBS, and permeabilized by acetone for 7 min. After being washed in PBS, the samples were blocked in PBS containing 0.3% Triton X-100, 10% donkey serum overnight, and then incubated in the same buffer with primary antibodies (CD31 antibody, 1:200, R&D systems, USA) for 2 nights. Secondary antibodies labeled with Alexa 594 (1:500; red fluorescence, Invitrogen) were used for 48 h after washing off the primary antibody with PBS. After washing each LG six times, the flattened samples were examined under a confocal microscope (Zeiss, Rossdorf, Germany). Images were processed using Image-pro Plus 6.0 (Media cybemetics, USA) and Adobe Photoshop (Adobe Systems, Inc., San Jose, CA, USA) software. CD31 positive staining quantification: the area of the positive staining pixel and corresponding tissue pixel in each field was measured with the pixel area as the standard unit. And the percentage of positive area is given by, positive area (%) = positive pixel area/tissue pixel area × 100.

### RNA Extraction and PCR Analysis

The mRNA expression of CD31, F5, Gas6, Fgg, and App in normal and diabetic LG was detected. Total RNA was extracted from collected mouse LG using NucleoSpin RNA kits (Takara, Dalian, China). cDNAs were synthesized using PrimeScript RT Master Mix (Takara). Real time PCR [Quantitative Reverse Transcription (qRT)-PCR] was carried out using ChamQ Universal SYBR qPCR Master Mix (Vazyme Biotech Co., Ltd., Nanjing, China) and the 7500 Real-Time PCR System (Applied Biosystems, Foster City, CA). The thermocycling conditions used were 30 s at 95°C followed by 40 two-step cycles (10 s at 95°C and 30 s at 60°C). The sequences of the primers used in this assay are listed in Table 1. The quantification data were analyzed with Sequence Detection System software (Applied Biosystems), using b-actin expression as an internal control.

**Table 1:** List of primers used for mRNA expression analyses.

**Gene name**	**Forward primer, 5′-3′**	**Reverse primer, 5′-3′**
GAPDH	CACTGAGCAAGAGAGGCCCTAT	GCAGCGAACTTTATTGATGGTATT
F5	ATACGCTGAAGTTGGGGACG	GGCATCATCCTTCCTCTCGG
Gas6	GGCTCAACTACACCCGAACA	GACAGTGACCGTGTGTTCCT
Fgg	ACGAGACAAGCATTCGGTATT	AGAACTGCTGCTTAGCTTTCAA
App	AAAGACAGACAGCACACCCTAA	ACTGGTTCATGCGCTCGTA
CD31	CAGAGCGGATAATTGCCATTC	TTCACAGAGCACCGAAGTACCA

### RNA-Sequencing and GO Analysis

The LG samples from type 2 diabetic mice or vehicles were collected with Trizol reagent. The mRNA was enriched by NEBNext^®^ Poly(A) mRNA Magnetic Isolation Module (New England Biolabs), and the produced RNA was used for construction Library, *via* KAPA Stranded RNA-Seq Library Prep Kit (Illumina). The prepared RNA-seq libraries were qualified using Agilent 2100 Bioanalyzer and quantified by the qPCR absolute quantification method. The sequencing was then performed using Illumina NovaSeq 6000. After quality control, the fragments were 5′, 3′-adaptor trimmed and filtered ≤ 16 bp reads with cutadapt software. The trimmed reads were aligned to a reference genome (source: genecode, version: GRCm38) with Hisat2 software. The transcript abundance for each sample was estimated with StringTie (v1.2.3), and the Fragments Per Kilobase of exon model per Million mapped fragments (FPKM) values for gene-level were calculated with R package Ballgown (v2.6.0). The differentially expressed genes (DEGs) analysis was also performed with Ballgown. Fold change (cutoff 1.5), *p*-value (cutoff 0.05), and FPKM (≥0.5 mean in one group) were used for filtering DEGs and transcripts. GO analyses of DEGs were performed *via* DAVID (6.8, http://david.ncifcrf.gov)(Gene Ontology, 2021). Gene count > 2 and *p* < 0.05 were set as the threshold.

### PPI Network Creation and Hub Gene Identification

PPI network of DEGs was constructed by STRING (11.0b; https://string-db.org/) with a combined score > 0.9 as the cut-off point (Kwon et al., 2018). Hub genes were identified using cytoHubba (Su et al., 2021), a plug-in of Cytoscape software (Cytoscape, 3.8.2), and significant modules in the PPI network were identified by MCODE 2.0.0 (Azad et al., 2018), another plug-in of Cytoscape software. The parameters of DEGs clustering and scoring were set as follows: MCODE score ≥ 4, degree cut-off = 2, node score cut-off = 0.2, max depth = 100, and k-score = 2.

### GeneMANIA

GeneMANIA (3.5.2, http://www.genemania.org) finds the most connected genes to a set of known genes (Franz et al., 2018), using a very large set of functional association data. Association data include protein and genetic interactions, pathways, co-expression, co-localization, and protein domain similarity. We used it to the weight that indicates the predictive value of hub genes and seeks the interactive functional network.

### CMap Analysis

The CMap (https://portals.broadinstitute.org/cmap) is an open resource that links disease, genes, and drugs by similar or opposite gene expression profiles (Yoo et al., 2019). CMap analysis is applied to predict potential small molecular compounds that can reverse altered expression of DEGs in cell lines. Mean < −0.4 and *p* < 0.05 were set as the screening criteria.

### Enzyme-Linked Immunosorbent Assay

The LG samples of diabetic and vehicle mice were collected, crushed, and separated by centrifugation. App and Fgg levels were determined by mouse enzyme-linked immunosorbent assay (ELISA) kits (SEB020Mu, SEC477Mu, Cloud-clone, China) according to the protocol of the manufacturer.

### Statistical Analysis

Data were analyzed by SPSS version 22.0 (IBM Corp., Armonk, NY, USA) and presented as the mean ± SD. Data normality was assessed by the Shapiro-Wilk test. Analysis of chi-squared test was performed to compare the gender and eye side of three groups of patients. Three groups of patients of age, duration of diabetes, tear secretion volume, longitudinal section of the LG, cross-section of the LG, PSV, RI, and PI were analyzed using one-way ANOVA and shown as mean ± SD. Repeated-measurement analysis of the unpaired Student's *t*-test was performed to compare the numerical values including blood glucose level, LG/total weight ratio, mice tear secretion, CD31+area/tissue field, expression of App and Fgg proteins, and relative expression of F5, Gas6, Fgg, App, and CD31 genes in LG from diabetic mice and vehicle group. The statistical analyses of DEGs were done in R software. The *p*-values in CMap analysis were analyzed in the CMap (https://portals.broadinstitute.org/cmap). A least-significant difference (LDS) was used as *post-hoc* analysis with *P* < 0.05 considered as significant.

## Results

### Comparison of LG Morphology and Hemodynamics Between Patients With Type 2 Diabetes and Healthy Controls

**Figure 2:**
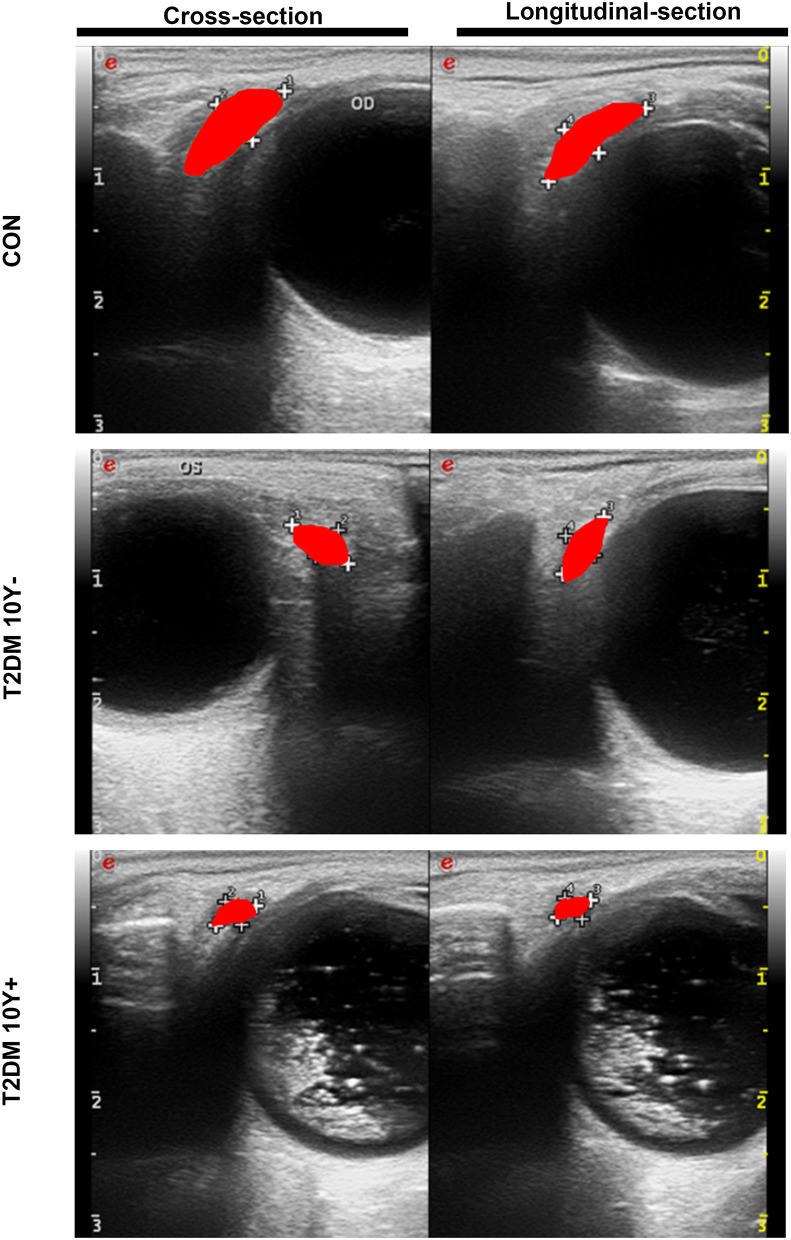
Morphological changes of LGs in patients with type 2 diabetes and control by CDFI. LGs from patients with T2DM and controls were measured by CDFI in two dimensions including the cross-section and longitudinal-section to demonstrate the morphological changes. The red area indicates the position and size of the LG. T2DM 10Y-, diabetic duration <10 years; T2DM 10Y+, diabetic duration more than 10 years; LG, lacrimal gland.

Lacrimal dysfunction is an important cause of morbidity in diabetic DE (Shih et al., 2017; Qu et al., 2021). To know the morphological and hemodynamic alterations of LG from patients with type 2 diabetes, the CDFI was performed for its advantage of not only demonstrating the shape of LGs but also providing real-time dynamic data of blood flow in LGs (Bilgili et al., 2005). As shown in Figure 2, Table 2, the tear secretion volume in the T2DM group decreased significantly, and the LG atrophied markedly in different dimensions in patients with diabetes as compared with healthy controls. However, the difference was not significant between patients with diabetes for <10 years and over 10 years. This suggested that, at the onset of type 2 diabetes, the LG had undergone an obvious pathological process that may continue to affect the growth and proliferation of the LG thereafter. Results of hemodynamics showed that no parameters displayed significant change compared with the control group within the early 10 years of diabetes. On the contrary, the RI and PI values increased significantly after 10 years of diabetes, indicating aggravated vascular resistance and the trend of insufficient blood supply or even ischemia in the LG after a long period of diabetic duration.

**Table 2:** Clinical characteristics of patients with type 2 diabetes and controls by color Doppler flow imaging (CDFI).

**Groups**	***N* (*n* = 30)**	**DM (10Y-, *n* = 30)**	**DM (10Y+, *n* = 30)**	***P*** **-values**		
				***N* vs. DM(10Y-)**	***N* vs. DM(10Y+)**	**DM(10Y-) vs. DM(10Y+)**
**Gender**						
Male	13	14	13	0.795	1.000	0.795
Female	17	16	17			
Age (years)	60.97 ± 12.63	59.00 ± 7.89	63.67 ± 9.00	0.45	0.28	0.07
DD (months)	0	4.47 ± 2.65	16.77 ± 6.92	<0.001	<0.001	<0.001
TS (mm)	9.17 ± 3.00	6.30 ± 4.22	6.33 ± 4.16	0.005	0.005	0.973
LG-L	1607.27 ± 456.39	1125.53 ± 383.15	1029.60 ± 294.01	<0.001	<0.001	0.34
LG-C	1297.77 ± 349.67	966.20 ± 404.34	853.73 ± 268.64	<0.001	<0.001	0.21
PSV(cm/s)	9.31 ± 3.13	8.17 ± 2.18	8.72 ± 2.82	0.11	0.41	0.44
PI	1.47 ± 0.42	1.72 ± 0.62	1.89 ± 0.58	0.081	0.003	0.215
RI	0.74 ± 0.10	0.76 ± 0.10	0.79 ± 0.09	0.44	0.02	0.11

*Variables are presented as mean ± SD. N, control group; DM10Y-, diabetic duration <10 years; DM10Y+, diabetic duration more than 10 years; OS, left eye; OD, right eye; DD, Duration of diabetes; TS, tear secretion; LG-L, longitudinal section of the lacrimal gland; LG-C, cross-section of the lacrimal gland; PSV, peak systolic velocity; RI, resistance index; PI, pulsatility index*.

### Histopathology and Vascular Distribution Alterations of LG in Type 2 Diabetic DE Mice Model

**Figure 3:**
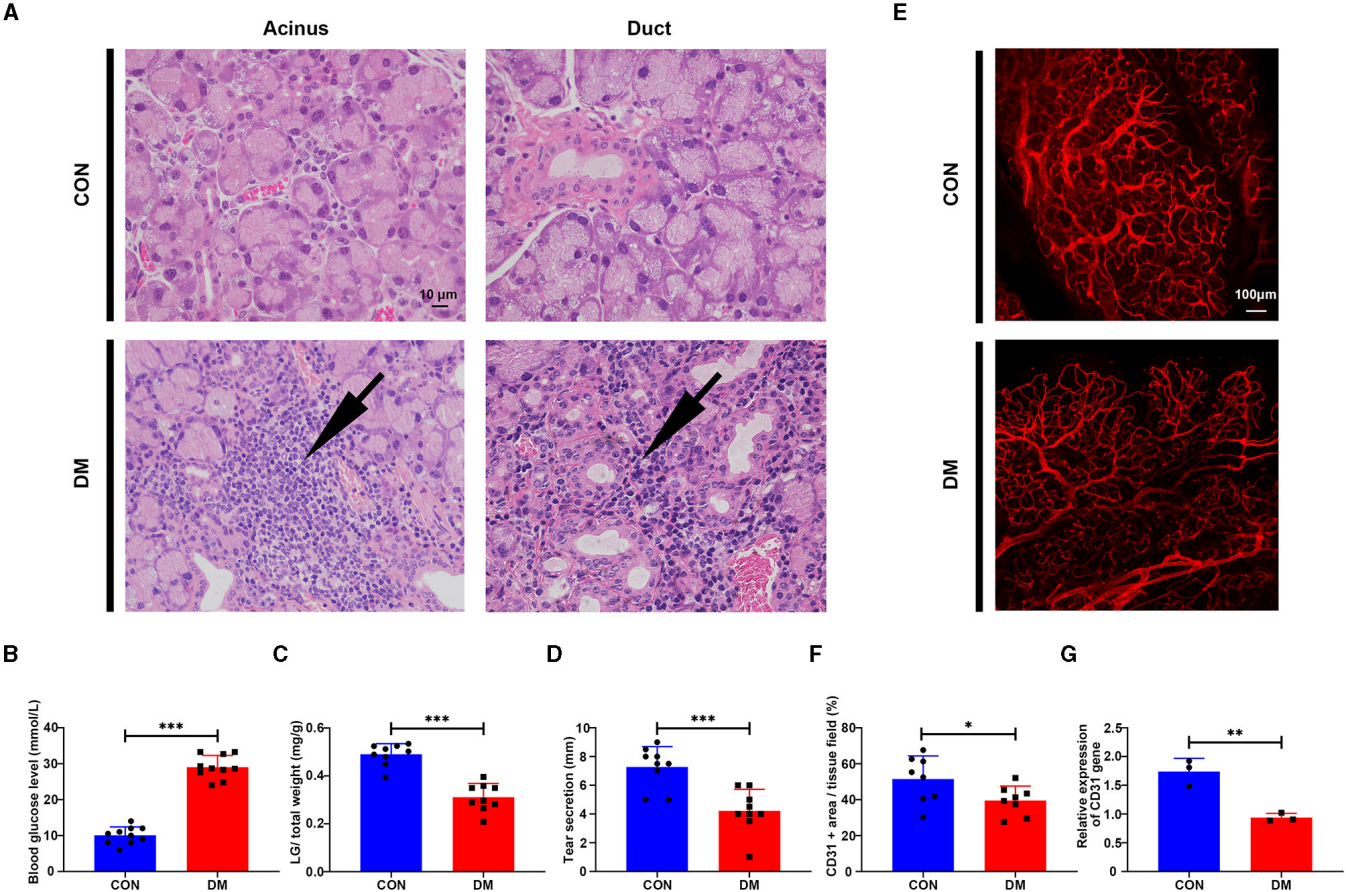
Histological change of lacrimal gland from type 2 diabetic mice and vehicles. **(A)** Hematoxylin-eosin (HE) staining of LG from type 2 diabetic mice and vehicles after 6 months of high-fat diet (HFD) and the last-time streptozotocin (STZ) injection. Arrow denotes the inflammatory infiltration. *N* = 3/group. **(B)** Blood glucose level after 6 months of HFD and STZ injection. *N* = 10/group. **(C)** LG/total weight ratio after 6 months of HFD and STZ injection. *N* = 9/group. **(D)** Tear secretion volume after 6 months of HFD and STZ injection. *N* = 9/group. **(E)** Measurement of vascular distribution in the lacrimal gland in the vehicle and type 2 diabetic mice after 6 months of HFD and STZ injection by immunofluorescent staining of CD31. *N* = 3/group. **(F)** Quantification of CD31^+^ area/lacrimal tissue field ratio from diabetic mice and vehicles. *N* = 8 fields/group. **(G)** Relative gene expression of CD31 in the lacrimal gland from diabetic mice and vehicles by PCR. *N* = 3/group. ^*^P < 0.05, ^**^P < 0.01, ^***^P < 0.001.

To feature the phenotypic and functional alterations of LG in type 2 diabetic DE, we established the type 2 diabetic mice model by a long-term HFD combined with STZ injection based on previous studies (Xing et al., 2021) and sacrificed them at the 24 weeks after the last time STZ injection. Hematoxylin-eosin (H&E), LG weighing, and phenol red thread tests were used to evaluate the histological change, LG size, and secretion function change in T2DM LG. The HE results showed that lacrimal acini atrophied and were transformed in diabetic mice, with severe inflammatory infiltrations gathered and aggravated around the acini and ducts (Figure 3A). The blood glucose level in T2DM mice was 29.06 ± 3.27 mmol/L compared with the level of 10 ± 2.36 mmol/L in the vehicle group (Figure 3B). The ratio of LG/total weight in the T2DM group was 0.31 ± 0.06 while it was 0.49 ± 0.04 in the vehicle group (Figure 3C). The results of phenol red thread tests showed tear secretion volume reduced to 4.22 ± 1.48 mm in T2DM mice, compared with the level of 7.28 ± 1.42 mm in the vehicle group (Figure 3D).

To test whether T2DM mice develop vasculature lesion in LG as diabetic patients, immunofluorescent staining and PCR analysis of CD31 (Angelini et al., 2018) were performed in mice LG samples. The results showed that the vessels (CD31 ^+^, red) in T2DM LGs were more sparsely distributed with less organized structures compared with the dense, aligned vasculature displayed by the healthy counterparts (Figures 3E,F). The gene expression level of CD31 was significantly downregulated with a level of 0.94 ± 0.07 in T2DM compared with 1.74 ± 0.23 in the vehicle group (Figure 3G). Taken together, these observations suggest that HFD+STZ treated mice model can successfully mimic the phenotype of patients with type 2 diabetes, exhibiting a reduction in tear secretion volume, lacrimal atrophy, and inflammatory infiltration. The sparsely distributed vasculature in T2DM mice may mimic the phenotypes of increased vascular resistance and the trend of insufficient blood supply in patients with T2DM.

### Identification of DEGs and GO Enrichment Analysis Related to Lacrimal Vasculopathy in T2DM

**Figure 4:**
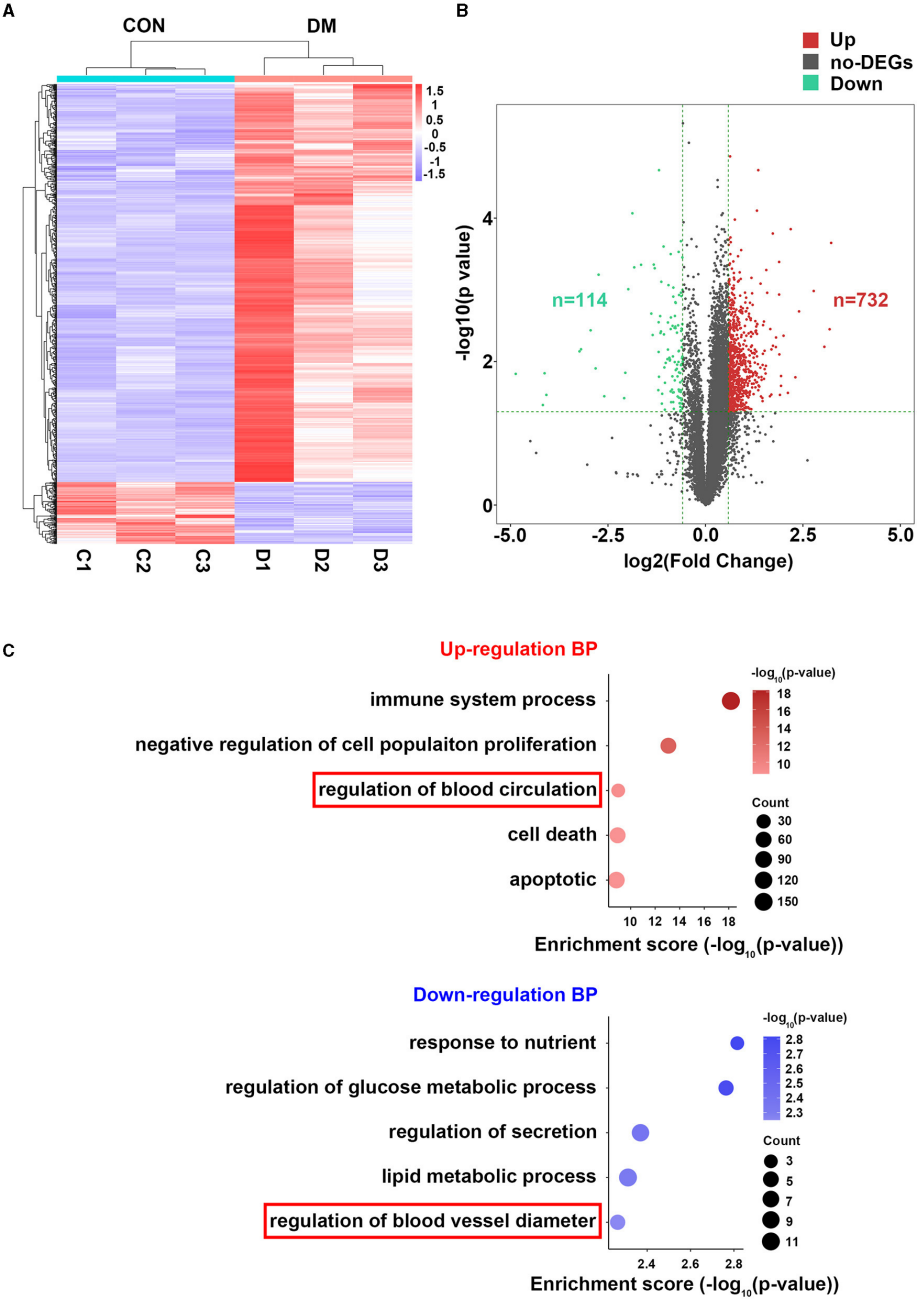
Bioinformatics analysis of LGs from type 2 diabetic mice and vehicles after 24 weeks of HFD and STZ treatments. **(A)** Heatmap showing the differentially expressed genes (DEGs) between LGs from diabetic mice and vehicles. *N* = 3/group. **(B)** Volcano plot showing all the DEGs between LGs from diabetic mice and vehicles. There were 732 upregulated genes and 114 downregulated genes in the diabetic group. **(C)** The GO analysis of enrichment of upregulated BP and downregulated BP in the form of bubble plot where dot size represents the number of genes overlapping with each pathway and the adjusted *p*-value is depicted by the color of the dot. The red frame denoting vasculature-related BPs. HFD, high-fat diet; STZ, Streptozotocin; DEG, differentially expressed gene; GO, gene ontology; BP, biological process.

To understand the mechanisms by which T2DM lacrimal vasculopathy was stimulated, we performed RNA-sequencing to identify differentially expressed genes (DEGs) between control and T2DM LGs. A total of 846 DEGs between T2DM and vehicle subjects were found as shown in the heatmap (Figure 4A). Among them, 732 genes were upregulated and 114 genes were downregulated (Figure 4B). The GO analysis showed enrichment of upregulated biological process (BP) including apoptotic process, cell death, immune system process, negative regulation of cell population proliferation, regulation of blood circulation, and downregulated BP including regulation of blood vessel diameter, regulation of secretion, response to nutrient, regulation of glucose metabolic process, and lipid metabolic process (Figure 4C). These findings from RNA-seq confirm the original observations in patients with type 2 diabetes and mice model that LG atrophy, secretion deficiency, inflammatory infiltration, and vasculature dysregulation are involved in diabetic lacrimal lesions. It is worth noting that App participates in the regulation of blood circulation, and Fgg participates in the regulation of blood vessel diameter, which is related to our following analyses.

### PPI Network and MCODE Analyses

**Figure 5:**
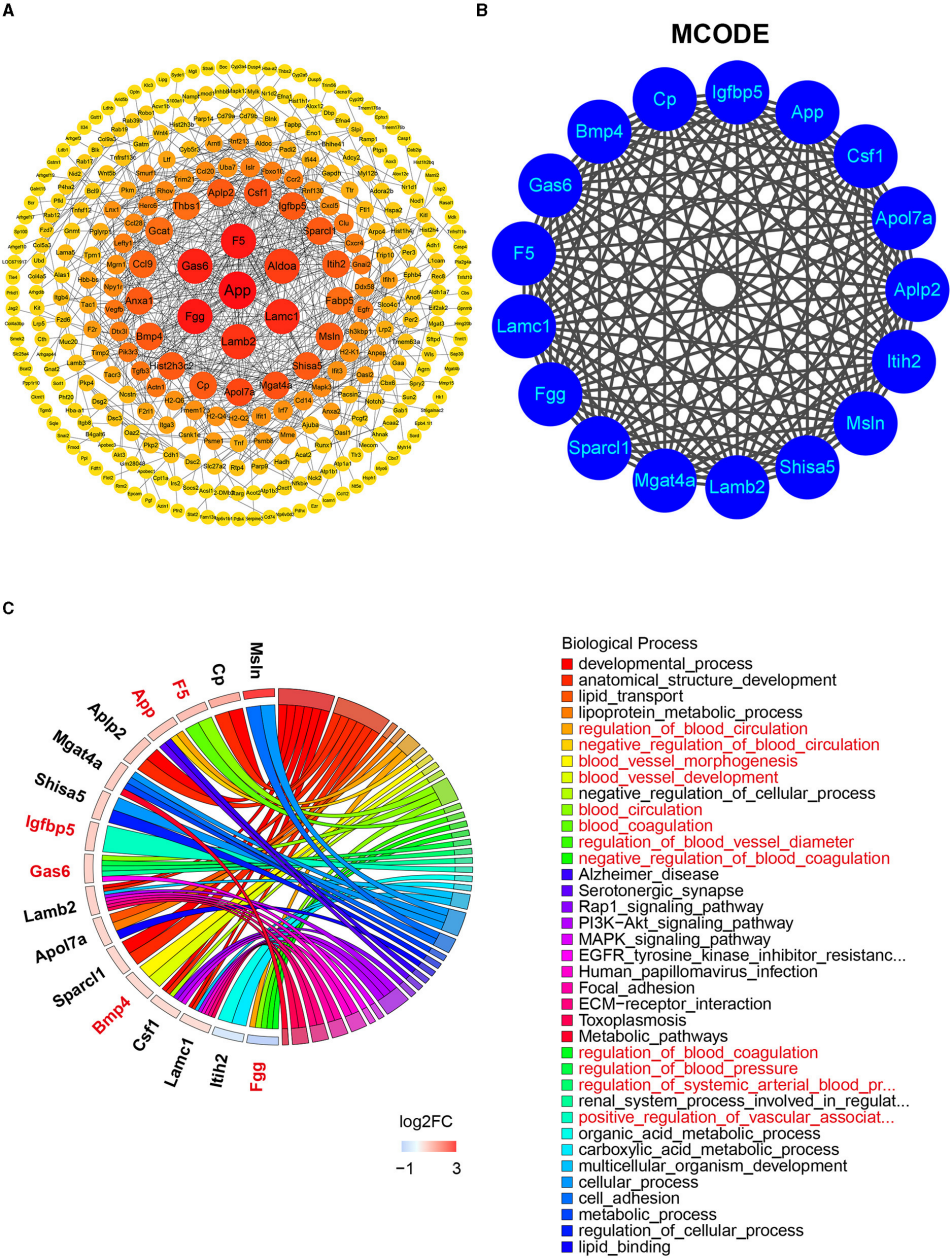
The PPI network, MCODE analysis, and involved biological process. **(A)** The PPI network was analyzed by String and Cytoscape software. The sizes and colors of the nodes are illustrated from big to small and red to yellow in descending order of degree values. **(B)** The most significant module was identified by MCODE including 17 related genes (score = 17). **(C)** The biological process related to the 17 genes by GO analysis. Red color denoting genes and BPs associated with vascular process/regulations. DEG, differentially expressed gene; PPI, protein–protein interaction; MCODE, molecular complex detection; GO, gene ontology.

To identify the most significant clusters of the DEGs, the PPI network of DEGs was constituted by STRING (confidence 0.9) and analyzed by Cytoscape. As shown in Figure 5A, the PPI network reflected the interaction between these potential targets. Seven targets highly related to the pathological process of diabetic LG lesion and dysfunction (DE), which were sorted according to the node degree, including App (degree = 42), F5 (Coagulation Factor V, degree = 25), Fgg (degree = 25), Gas6 (Growth Arrest Specific 6, degree = 24), Lamc1 (Laminin subunit gamma 1, degree = 22), Lamb2 (Laminin subunit beta 2, degree = 21), and Aldoa (Aldolase, Fructose-Bisphosphate A, degree = 20). The most significant module (score = 17) was recognized by MCODE (node score cutoff: 0.2, K-core: 2, degree cutoff: 2, maximum depth: 100), a plugin of Cytoscape (Figure 5B), with 17 genes (App, Csf1, Apol7a, Aplp2, Itih2, Msln, Shisa5, Lamb2, Mgat4a, Sparcl1, Fgg, Lamc1, F5, Gas6, Bmp4, Cp, and Igfbp5) involved. The BP related to these genes was present in Figure 5C. Among the 17 genes in the module, six genes (App, Gas6, F5, Fgg, Bmp4, and Igfbp5) were associated with vascular process/regulations, including regulation of blood circulation, blood vessel morphogenesis, blood vessel development, regulation of blood coagulation, and regulation of blood vessel diameter.

### Identification of Hub Genes and Validation

**Figure 6:**
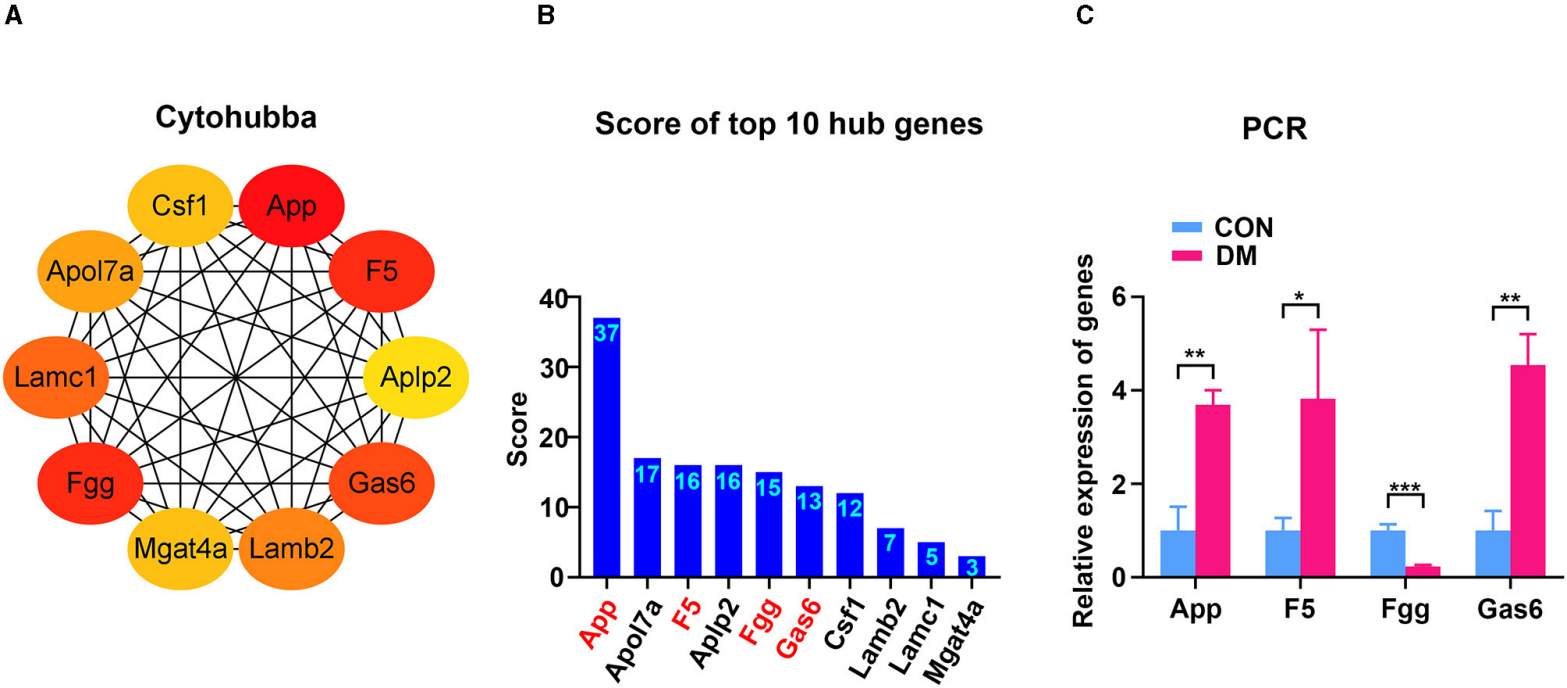
Identification and confirmation of the hub genes. **(A)** Ten hub genes were identified by cytoHubba. Four hub genes (App, F5, Fgg, and Gas6) were detected by both MCODE and cytoHubba. **(B)** The concrete scores of the hub genes. **(C)** Relative expression of the four hub genes above by PCR. *N* = 3/group. ^*^P < 0.05, ^**^P < 0.01, ^***^P < 0.001. MCODE, molecular complex detection; PCR, polymerase chain reaction.

**Figure 7:**
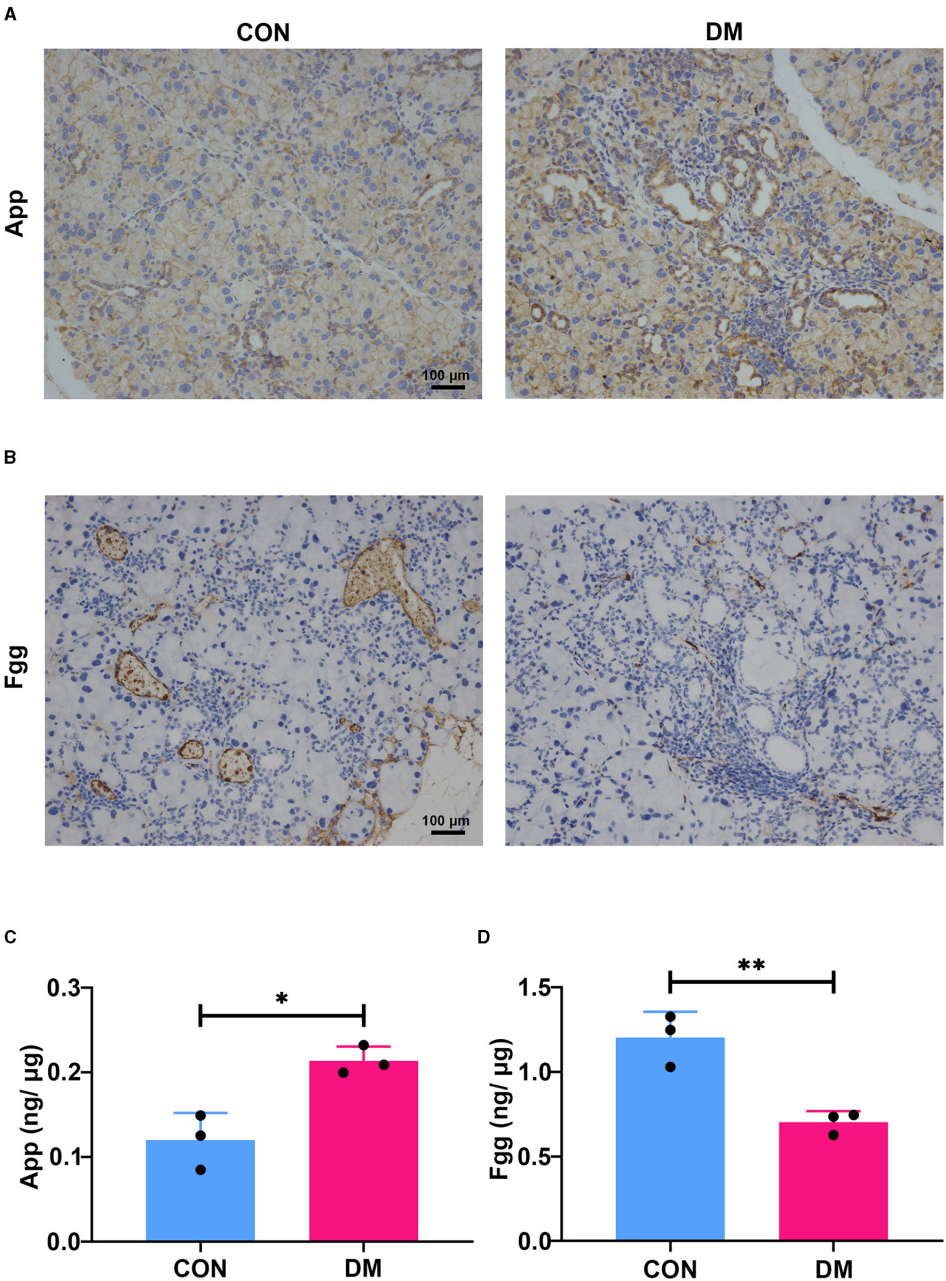
Expression of App and Fgg in diabetic lacrimal glands. **(A)** The representative images of immunochemistry staining for App in T2DM and vehicle LGs at 6-month diabetic period. The App was mainly expressed at ducts, vessels, and acini. The diabetes group showed obvious expression aggravation. Scale bar: 100 μm. **(B)** The representative images of immunochemistry staining for Fgg in T2DM and vehicle LGs at a 6-month diabetic period. Fgg was expressed in vessels and concentrated at a vascular endothelium. The diabetes group showed obvious expression reduction. Scale bar: 100 μm. **(C)** The protein level of App in diabetic and age-matched vehicle LGs by ELISA (*N* = 3/group). **(D)** The protein level of Fgg in diabetic and age-matched vehicle LGs by ELISA (*N* = 3/group). **(A–D)**, data are representative of three independent experiments (*n* = 3/group). C&D, Data were presented as the mean ± SEM. ^*^P < 0.05, ^**^P < 0.01. T2DM, type 2 diabetes; LG, lacrimal gland.

To identify the hub gene in the DEGs, cytoHubba, another plug-in Cytoscape was applied. All the gene codes and edges were calculated. The top 10 genes were identified as hub genes (Figure 6A). The concrete scores of these hub genes were shown in Figure 6B. We have noticed that all the hub genes were included in the most significant module above, and four hub genes (App, F5, Fgg, and Gas6) related to vascular regulation were identified repeatedly. To verify the gene expression level of these four hub genes, PCR measurement was performed to confirm the expression (Figure 6C). Consistent with the results of RNA-seq, the gene expression of App, F5, and Gas6 were significantly upregulated in the T2DM group, while expression of Fgg was significantly downregulated. We chose the most significant upregulated gene (App) and downregulated gene (Fgg), which were related to vasculature lesion and examined their protein level by ELISA and immunochemistry. The results were consistent with our former observations. In T2DM LG, which expressed low amounts of Fgg (Figures 7B,D), App was expressed at high levels and mainly located at lacrimal ducts, vessels, and acini (Figures 7A,C).

### GeneMANIA Analysis

**Figure 8:**
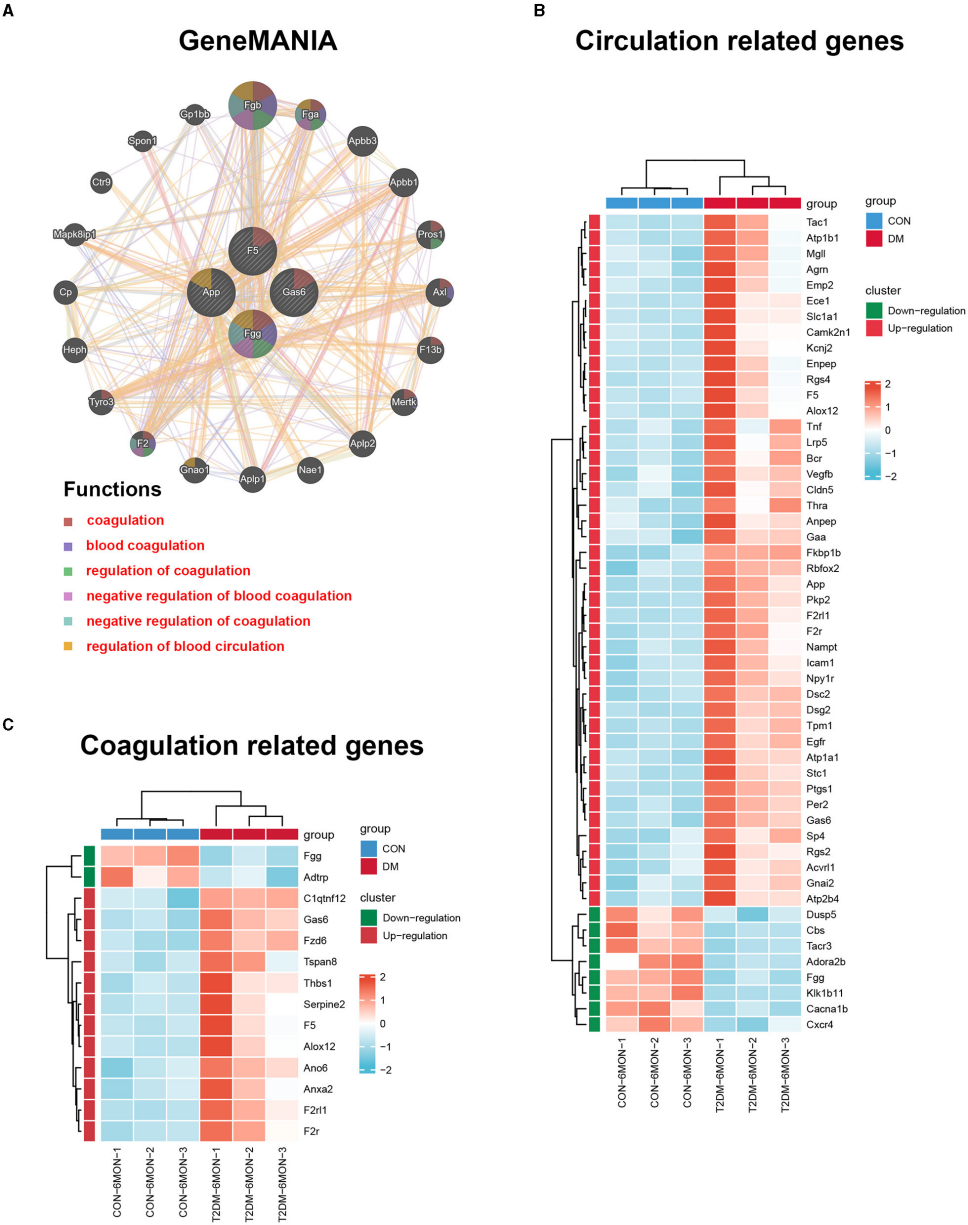
GeneMANIA analysis and the expression heatmaps of the functionally related genes. **(A)** GeneMANIA analysis was performed to find genes with shared properties and similar functions to the four hub genes above and the interactive functional association network. Interactive functional association networks showed a connection with the regulation of coagulation and circulation. **(B)** The differential expression of circulation-related genes in T2DM and control groups. **(C)** The differential expression of coagulation-related genes in T2DM and control groups. The data were transferred to the Z-score of FPKM by each row and clustered based on the Euclidean distance (*n* = 3/group).

To further investigate genes with shared properties and similar functions to the four hub genes identified above, and to display their interactive functional association network, GeneMANIA analysis was performed. The results revealed 20 kinds of molecules that most associated with the four hub genes. The functions of the hub genes and their associated molecules (such as Fgb, Fga, Pros1, Axl, F13b, Mertk, Gnao1, F2, and Tyro3) were primarily related to the regulation of coagulation and regulation of blood circulation (Figure 8A). To observe the expression of all genes enriched in the circulation and coagulation-related BP, we extracted data from GO analysis. The results showed that 44 genes were upregulated and 8 genes were downregulated in the circulation-related process (Figure 8B), while 12 genes were upregulated and 2 genes were downregulated in the coagulation-related process (Figure 8C).

### CMap Analysis

To search for potential small molecular compounds to reverse altered expression of DEGs, CMap analysis was performed. The most three significant small molecular compounds were Thapsigargin, Tanespimycin, and Lomustine (Table 3). Among the potential small molecular compounds, 5/20 (25%) compounds were related to the treatment of the diabetic vascular disorder, including Disulfiram, Bumetanide, Ambroxol, Chenodeoxycholic acid (CDCA), and genistein.

**Table 3:** List of the 20 most significant small molecular compounds provided by connectivity map (CMap) analysis to reverse altered expression of differentially expressed genes (DEGs) in cell lines.

**CMap name**	**Mean**	**Enrichment**	***P***	**Percent non-null**	**Structure**
Thapsigargin	−0.841	−0.989	<0.00001	100	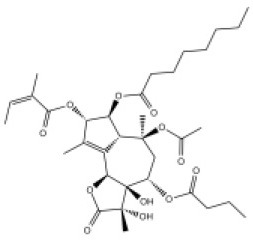
Tanespimycin	−0.228	−0.382	<0.00001	59	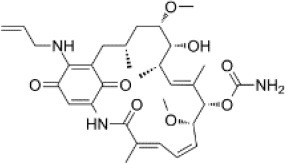
Lomustine	−0.672	−0.903	0.00016	100	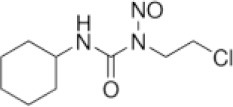
Disulfiram	−0.626	−0.838	0.0003	100	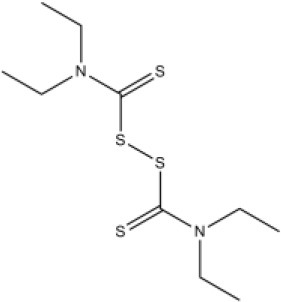
Meclocycline	−0.558	−0.874	0.00054	100	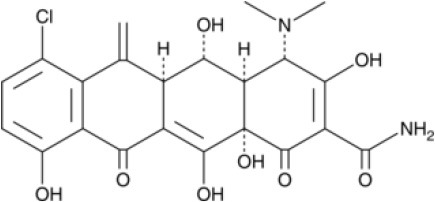
Diethylstilbestrol	−0.564	−0.728	0.00075	83	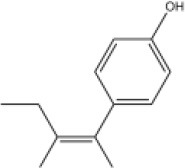
Semustine	−0.639	−0.83	0.00157	100	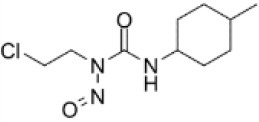
Lanatoside C	−0.48	−0.704	0.00157	83	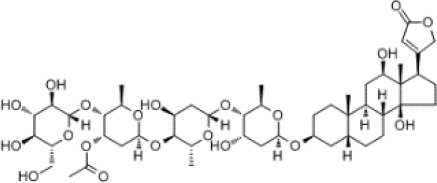
Metrizamide	−0.528	−0.815	0.00211	100	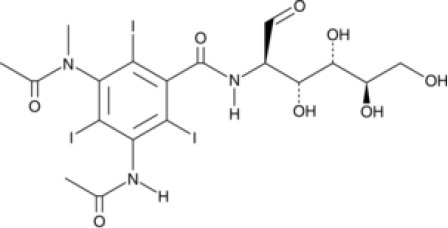
Bumetanide	−0.459	−0.809	0.00259	100	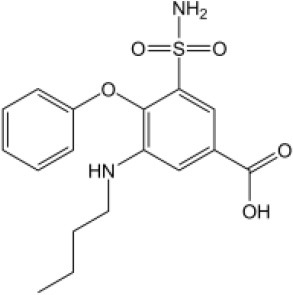
Geldanamycin	−0.3	−0.454	0.00268	66	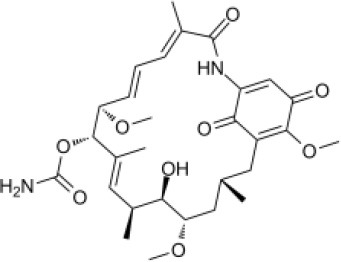
Ambroxol	−0.642	−0.803	0.00292	100	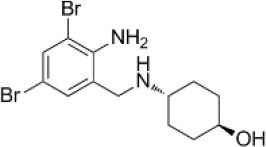
Trimethobenzamide	−0.312	−0.729	0.00308	60	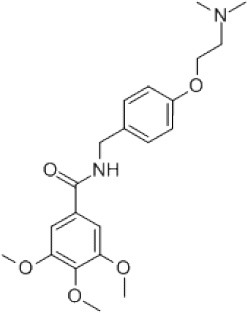
Chenodeoxycholic Acid	−0.564	−0.8	0.0031	100	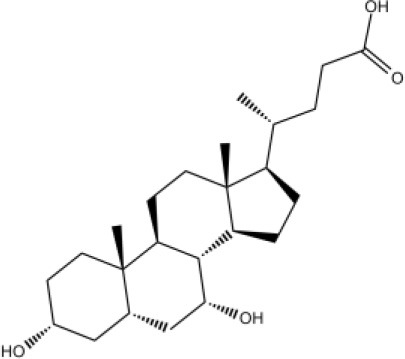
Pilocarpine	−0.547	−0.798	0.00332	100	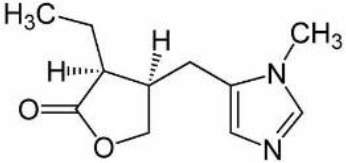
Genistein	−0.194	−0.421	0.00332	52	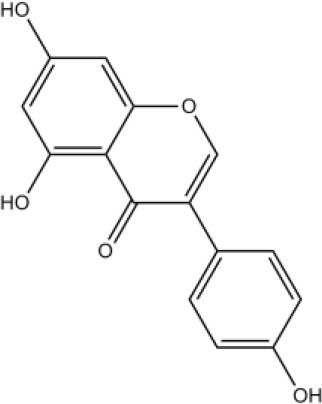
Cobalt Chloride	−0.528	−0.857	0.00575	100	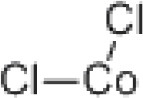
Anisomycin	−0.56	−0.758	0.0071	75	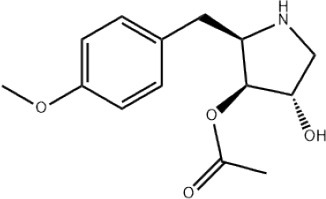
Estrone	−0.444	−0.752	0.00764	75	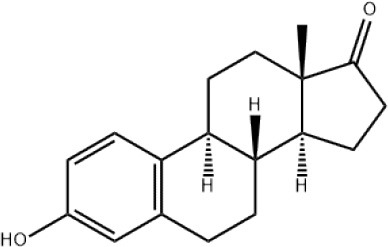
Theobromine	−0.452	−0.724	0.0118	75	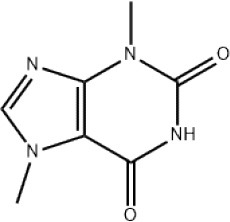

## Discussions

Although the morbidity of diabetes-related complications has reduced over the past decades, patients with diabetes still have a significantly increased risk for vascular complications as compared with individuals without diabetes (Low Wang et al., 2016; Ling et al., 2020). In this study, we explored whether vasculature dysfunction is involved in the lacrimal lesion to induce DE in type 2 diabetes by targeting vascular-related genes. Collectively, our study demonstrated that four critical genes and unbalanced circulation and coagulation signaling pathways were associated with lacrimal vasculature dysfunction in T2DM DE by bioinformatic methods.

Dry eye is one of the major complications of DM on the ocular surface, which is closely linked with lacrimal lesions (Shih et al., 2017; Jiao et al., 2020; Qu et al., 2021). Diabetic angiopathy (DA) is another common and severe complication of DM and is characterized by high morbidity, early-onset, and rapid progression (Packer, 2018). In diabetes, there is also impaired collateralization of vascular ischemic beds (Fadini et al., 2005), but phenotypes and mechanisms that involved lacrimal vasculature dysregulation in diabetes remain elusive. In this study, we evaluated the morphology and hemodynamics of LGs in patients with T2DM by CDFI. As a non-invasive imaging technique without radiations or high magnetic field exposure, CDFI can be used to measure the lacrimal morphology and hemodynamics (Lecler et al., 2017). We found that patients with diabetes exhibited prominently lacrimal atrophy in all dimensions along with RI and PI parameters elevation. RI and PI are vital parameters for characterizing the arterial waveform and vascular resistance (Halpern et al., 1998; Bude and Rubin, 1999; Grenier et al., 2001; Heine et al., 2005). Increased vascular resistance along with tissue atrophy in diabetes indicated the occurrence of the hypoxia-ischemic syndrome in the tissue endings (Liu et al., 2017; Nilsson et al., 2020), which is a common mechanism of diabetic vascular complications (Fadini et al., 2010; Mudaliar et al., 2021).

To further explore the mechanism of vascular complication in T2DM LG, we established the T2DM mice model by STZ+HDF treatments (a mice model progressively developed T2DM, which mimicked that seen in patients with diabetes, as opposed to genetic models that rapidly develop T2DM spontaneously) (Kleinert et al., 2018) and affirmed the appearance of diabetic DE phenotype. Consistent with patients with T2DM, we found lacrimal atrophy and vasculature lesion with sparse vascular distribution in T2DM mice as compared with the healthy counterparts. Even though we did not assess the vascular resistance parameters on the mice model, the sparse vasculature distribution may also represent a peripheral blood supply decrease and indicate an ischemia-like phenotype (Yagihashi et al., 2011). Indeed, patients with DM and a prior ischemic vascular event are at an especially high risk of recurrent events (Mann et al., 2018; Verma et al., 2018; Rawshani et al., 2019). Depending on the severity, ischemia and ischemia/reperfusion injury (IRI) may lead to ischemic necrosis, which further leads to progressive injury, fibrosis, and atrophy irreversibly (Holderied et al., 2020). This partly explains why the vasculature changes in the LGs of diabetes are accompanied by tissue and acinus atrophy. However, the exact mechanism of lacrimal ischemia in type 2 diabetes remains elusive.

With further RNA-seq analysis, 846 DEGs were found between LGs from T2DM mice and vehicles. Functional enrichment analysis showed upregulation of immune system process, cell death, apoptosis process, blood circulation and downregulation of blood vessel diameter, secretion, response to nutrient, glucose metabolic process, and lipid metabolic process. All these terms were consistent with the phenotypes as seen in patients with T2DM and mice model, including inflammatory cell infiltration, tissue atrophy, abnormality of vascular distribution, high vascular resistance, tear secretion deficiency, high glucose levels, and so on. Most of these phenotypes were also seen in the mice model of T1DM (Jiao et al., 2020; Qu et al., 2021). To further explore the mechanism on lacrimal ischemia in T2DM, PPI network, MCODE, and cytoHubba analyses were applied as their function of finding the core module and hub genes among all the DEGs. We thus found four hub genes (App, F5, Fgg, and Gas6) that were closely related to vascular regulation and diabetes. Meakin Paul J reported that elevated App was linked with vascular disease development in obesity and diabetes (Meakin et al., 2020). Lodigiani C et al. revealed that there were connections between the F5 gene variant, diabetes and atherothrombosis and other vascular complications (Lodigiani et al., 2009). Activated protein C (APC) resistance is associated with the F5 Leiden mutation, and it is the most common risk factor for venous thrombosis. Omarova F revealed that Fgg can increase plasma APC sensitivity and reduction of Fgg might decrease the basis for pharmacologic interventions to counteract APC resistance (Omarova et al., 2014). Lee CH reported that the plasma protein growth arrest-specific 6 (Gas6) is important to the inflammatory process and involved in the development of diabetic renal and vascular complications (Lee et al., 2012). Up to now, there is still no report on the correlation between the genes of App, F5, Fgg, and Gas6 with diabetic lacrimal lesions. The GeneMANIA further revealed that the functions of the four hub genes above and their associated molecules (such as Fgb, Fga, Pros1, Axl, F13b, Mertk, Gnao1, F2, and Tyro3) were primarily related to the regulation of circulation and coagulation. In addition, we found that 44 genes were upregulated in the circulation-related BP and 12 genes were upregulated in the coagulation-related BP. This is interesting for these two BPs are both connected with ischemia lesions (Dong et al., 2019; Wilbs et al., 2020). Activation of coagulation factors is a driving force of IRI in acute stroke (Stoll and Nieswandt, 2019). To counterpart this trend, angiogenesis and other pathways to promote blood circulation are required for tissue remodeling/repair following ischemia (Meng et al., 2018).

In the present study, we found several potential small molecular compounds to reverse the altered expression of the DEGs, which might improve lacrimal lesions and vasculature dysfunction in T2DM. It was reported that the therapy of an ophthalmic *in situ* gel formulation incorporating disulfiram (DIS) nanoparticles (Dis-NPs/ISG) might contribute to restore retinal dysfunction in diabetic retinopathy (DR), a diabetic vascular lesion (Deguchi et al., 2020). Bumetanide also has been demonstrated to play an important role in antidiabetic activity with low *in vitro* cell toxicity through antiinflammatory effects (Navas et al., 2020) and can reduce diabetic vascular dysfunction by suppression of angiogenesis (Topper et al., 1997; Guzel et al., 2020). Ambroxol can significantly inhibit proinflammatory cytokines, reduce lung inflammation, and accelerate recovery by reducing vascular permeability in acute lung injury (Su et al., 2004). However, whether ambroxol could improve diabetic vasculopathy remains unclear. CDCA was reported to promote eNOS phosphorylation and NO production that contributes to its vasorelaxant effect (Wang et al., 2017). Babu PV reported that genistein can improve diabetes-caused vascular inflammation by suppressing diabetes-induced adhesion of monocytes to endothelial cells and endothelial secretion of adhesion molecules (Babu et al., 2012). However, the other small molecular compounds have not been reported to have the function to reverse vasculature dysfunction or diabetes. The CMap data set uses cellular responses to mostly Food and Drug Administration (FDA) approved drugs to find relationships between small molecules, genes, and diseases (“connectivity mapping”), despite the need for further research of drugs application (Kohonen et al., 2017). All these small molecular compounds could be explored as novel therapeutic targets to treat vasculature dysfunction, diabetes, and related metabolic diseases.

Overall, we may conclude that upregulation of App, F5, Gas6, and downregulation of Fgg may induce vasculature dysfunction in T2DM by modulating the regulation of circulation and coagulation pathways. Our results provide important novel information to further understand the effects of vasculature dysfunction in T2DM LG lesions and predict the potential therapeutic molecular compounds to reverse the gene expression in the disease. However, further work needs to address which of these possibilities targets and medications apply and whether they all act in unison.

## Data Availability Statement

The original contributions presented in the study are included in the article/Supplementary Material, further inquiries can be directed to the corresponding author/s. All RNA-seq data presented in this study are deposited in the Gene Expression Omnibus under the accession number GSE179231.

## Ethics Statement

The studies involving human participants were reviewed and approved by Ethics Committee of Qingdao Eye Hospital. The patients/participants provided their written informed consent to participate in this study. The animal study was reviewed and approved by Experimental Animal Ethics Committee of Shandong first medical University.

## Author Contributions

JX, BZ, MD, MZ, and HW collected and analyzed the data. JX, BZ, and SD wrote the manuscript. DL, LX, JX, SD, BZ, QZ, and YD designed the research. All authors contributed to the article and approved the submitted version.

## Conflict of Interest

The authors declare that the research was conducted in the absence of any commercial or financial relationships that could be construed as a potential conflict of interest.

## Publisher's Note

All claims expressed in this article are solely those of the authors and do not necessarily represent those of their affiliated organizations, or those of the publisher, the editors and the reviewers. Any product that may be evaluated in this article, or claim that may be made by its manufacturer, is not guaranteed or endorsed by the publisher.
